# Different Stages of Alveolar Bone Repair Process Are Compromised in the Type 2 Diabetes Condition: An Experimental Study in Rats

**DOI:** 10.3390/biology9120471

**Published:** 2020-12-16

**Authors:** Letícia Pitol-Palin, Fábio Roberto de Souza Batista, Pedro Henrique Silva Gomes-Ferreira, Gabriel Mulinari-Santos, Edilson Ervolino, Francisley Ávila Souza, Dóris Hissako Matsushita, Roberta Okamoto

**Affiliations:** 1 Department of Diagnosis and Surgery, Araçatuba Dental School, São Paulo State University Júlio de Mesquita Filho, Araçatuba 16015-050, Brazil; fabiorsbatista@gmail.com (F.R.d.S.B.); pedroferreirabmf@gmail.com (P.H.S.G.-F.); gabriel_mulinari@hotmail.com (G.M.-S.); francisley.avila@unesp.br (F.Á.S.); 2 Department of Basic Sciences, Araçatuba Dental School, São Paulo State University Júlio de Mesquita Filho, Araçatuba 16066-840, Brazil; e.ervolino@unesp.br (E.E.); doris.hissako@unesp.br (D.H.M.); roberta.okamoto@unesp.br (R.O.)

**Keywords:** diabetes mellitus, high-fat diet, streptozotocin, tooth socket, bone density

## Abstract

**Simple Summary:**

Type 2 diabetes (T2D) affects more than 90% of all patients diagnosed with diabetes, and among its risk factors, unhealthy eating habits are worth mentioning. With the notorious increase in the incidence of diabetic patients, there has also been an increase in surgical complications in dentistry, so this work presents a study model that mimics the T2D condition in rats, where animals receive a diet composed of foods rich in sugar and fat equivalent to the poor diet of the current population. The animals were submitted to dental extraction to perform analyzes at different stages of the alveolar bone. It is important to highlight that with the development of this experimental model it will be possible to simulate different conditions that are observed in clinics and in consequence and improve the characterization of the cellular responses involved in this complex condition of T2D. The scientific evidence presented in this study shows that T2D prolongs the local inflammatory process, which impairs the organization and maturation of collagen fibers, delaying bone formation and bone turnover. This fact implies in a series of disorders in dental practice, that would need to compensate in other ways, either with systemic medications or local therapies.

**Abstract:**

The aim of this study was to analyze the stages of the alveolar bone repair in type 2 diabetic rats evaluating the mechanism of mineralization and bone remodeling processes after dental extraction. Forty-eight rats were divided into normoglycemic (NG) and type 2 diabetes (T2D) groups. The upper right incisor was extracted and after 3, 7, 14 and 42 days the animals were euthanized. The following analyses were performed: immunolabeling against antibodies TNFα, TGFβ, IL6, WNT, OCN and TRAP, collagen fibers maturation, microtomography and confocal microscopy. Data were submitted to statistical analysis. The immunolabeling analysis showed that the T2D presented a more pronounced alveolar inflammation than NG. Labeling of proteins responsible for bone formation and mineralization was higher in NG than T2D, which presented greater resorptive activity characterized by TRAP labeling. Also, T2D group showed a decrease in the amount of collagen fibers. Micro-CT analysis showed that T2D causes a decrease in bone volume percentage due to deficient trabecular parameters and higher porosity. The T2D bone dynamics show a loss in bone remodeling process. T2D prolongs the local inflammatory process, which impairs the organization and maturation of collagen fibers, delaying bone formation that generates impact on mineralization and bone turnover.

## 1. Introduction

Type 2 diabetes (T2D) affects more than 90% of all diabetic patients [1]. By 2040 there will be an estimated 642 million diabetic people worldwide [2]. This condition is characterized by the presence of chronic hyperglycemia, dyslipidemia, hypertension and endothelial dysfunction [3], as well as serving as a predisposing factor for metabolic syndrome [4] and cardiovascular diseases [5]. Consequently, it leads to impacts on various body tissues, such as the kidneys, nerves, eyes, blood vessels and bones [3,4,6]. Thus, it impairs at collagen and calcium metabolism, with delayed bone repair, increased risk of osteopenia and fractures in adults older than 65 years [6,7]. Among the main risk factors for T2D are obesity and an unhealthy lifestyle with foods high in sugar, fat and sodium in combination with a lack of physical activity [3,4].

The cafeteria diet is the closest equivalent to the industrially processed high-energy food diet found in supermarkets. This diet simulates the main obesogenic characteristics of human diets, as well as predisposes insulin resistance condition and in consequence, the T2D development [8,9]. In addition, weight gain related to obesogenic diets increases the expression of proteins associated with the inflammatory process [10]. There are several studies for T2D models [10], like genetically modified mice [11], alloxan induction [8] and streptozotocin (STZ) models [3,12,13]. STZ is an antineoplastic agent with destructive action of β-pancreatic cells [14,15,16]. As malnutrition habit consists on a factor that may predispose T2D [9], reflecting most of the patients affected by this condition. It is reasonable to use a model that allow to mimic this situation, with the association of cafeteria diet, inducing an insulin resistance condition and the streptozotocin low dose, attacking β-pancreatic cells.

Bone also responds to the effects of T2D, because high glucose levels inhibit the differentiation and function of the MC3T3-E1 osteoblastic strain [17]. Insulin is known to have anabolic action on bone tissue, so patients with type 1 diabetes tend to have impaired bone density [18]. However, chronic hyperglycemia affects collagen metabolism, contributing to decreased bone quality [2,19]. Long bones of diabetic patients have a greater predisposition to fractures due to decreased bone density [20]. The oral cavity responds to type 1 diabetes with bone loss due to periodontal disease, in addition to the risk of infections and delayed post-exodontic tissue repair [19,21,22,23]. Alveolar bone repair compared to T2D is not widely described in the literature, as well as cellular mechanisms throughout the repair phases. With the notorious increase in the incidence of patients diagnosed with T2D and in how this fact generates concern for public health, studies that bring answers on how specialized tissues respond to this metabolic disorder become necessary. Thus, the aim of this study was to analyze the stages of the alveolar bone repair in type 2 diabetic rats evaluating the mechanism of mineralization and bone remodeling processes after dental extraction.

## 2. Materials and Methods

### 2.1. Animals

The ethics of the study was approved by the Research Ethics Committee of Araçatuba Dental School, under number 00385-2017 (on 19 April 2017). The study was presented following the Animal Research N3CR guidelines for Reporting of In Vivo Experiments (ARRIVE) guidelines [24]. Forty-eight 250 g weight and 3-month-old male Wistar rats (*Rattus norvegicus albinus*) were divided in two groups (*n* = 24, in each group): Normoglycemic (NG) and type 2 diabetes (T2D). The animals were identified by numbers and randomly separated by Microsoft Office Excel software (Microsoft, Redmond, WA, USA), respecting 1:1 allocation rate for each group.

The animals were kept in cages in a stable temperature environment (22 °C ± 2 °C, light control cycle 12 light h, 12 h dark), balanced diet (NUVILAB, Paraná, Brazil, 1.4% Ca and 0.8% P + water ad libitum). After one week, the T2D group were feed with a cafeteria diet (defined below), water with sugar and water ad libitum, and the NG group followed with the balanced diet (NUVILAB, Paraná, Brazil, 1.4% Ca and 0.8% P + water ad libitum). The animals were kept in the Animal Facilities until respective euthanasia at 3, 7, 14 or 42 days after dental extraction.

### 2.2. Diabetes Induction

Throughout the experiment, the T2D animals were feed with cafeteria diet [4,9], which shows that cafeteria diet causes insulin resistance, obesity and can lead to metabolic syndrome. In addition, the cafeteria diet mimics the poor diet consumed by world population. NG animals were fed conventional ration throughout the experiment. Each animal was weighed daily with 30 g of food, with stuffed crackers, wafer crackers, corn chips and also a bottle of water with a concentration of 12% of sucrose (Table 1).

After three weeks of cafeteria diet (day 21), the T2D animals were anesthetized by intramuscular infiltration of xylazine hydrochloride (Dopaser^®^—Laboratórios Calier do Brazil Ltd.a., Osasco, Brazil) and ketamine hydrochloride (Vetaset^®^—Fort Dodge Animal Health Ltd.a., Campinas, Brazil) at the dosage indicated by the manufacturer. Asepsis of the scrotum region was performed with 70% alcohol, and in the T2D rats were injected with low-dose STZ (Sigma^©^, Merck KGaA, Darmstadt, Germany and/or its affiliates) dissolved in vehicle (sodium citrate solution 0.1 M, pH = 4.5) via the penile vein (35 mg/kg) [3,13]. NG rats received the vehicle through the penile vein.

**Figure 1:**
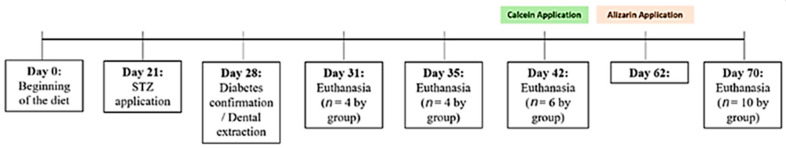
Experimental model timeline. Beginning of the diet = day 0; STZ application = day 21; Diabetes confirmation/dental extraction = day 28; Fluorochromes application = days 42 and 62; and Euthanasia = at days 31, 35, 42 and 70.

Diabetes confirmation was made one week after T2D induction (day 28), following the criteria: glucose concentration greater than 198 mg/dL after 120 min of oral glucose solution [3] with a concentration of 12% of sucrose. (Figure 1).

### 2.3. Dental Extraction

After diabetes confirmation (at day 28), all animals were anesthetized and the right upper incisor of all animals was extracted, using specially adapted instruments for this purpose [25]. The gingival mucosa was sutured with polyglactin 910 (Vicryl 4.0—Johnson & Johnson, New Brunswick, NJ, USA). In the immediate postoperative period, 0.2 mL of a veterinary antibiotic (penicillin with streptomycin 1,200,000 IU) was used intramuscularly in all animals. (Figure 1).

### 2.4. Fluorochromes Application

Fourteen days after dental extraction, the animals designated for euthanasia at 42 days received an intramuscular injection of calcein (20 mg/kg). After 20 days, the same animals received an intramuscular injection of alizarin red (20 mg/kg) [26,27,28]. Fluorochromes are chemical compounds that bind to calcium at the time of precipitation in the bone matrix. Therefore, the extent of fluorochrome labeling represents the amount of calcium precipitation, thus allowing measurement of bone formation. Another aspect to consider is the period in which the fluorochromes were injected; calcein (green) represents the old bone and alizarin (red) represents the new bone. (Figure 1).

### 2.5. Glycemia and Body Weight

Starting the cafeteria diet, the animals glycemia and body weight were measured weekly with an Accu-Chek Performa glucose-meter (Roche Diagnostics, Mannheim, Germany) and a digital scale. The periods analyzed in this study were: beginning of the diet, end of three weeks of diet, diabetes confirmation/dental extraction, and euthanasia.

To perform the glycemic evaluation, the animals were fasted for 2 h after consuming the oral solution containing glucose (12% glucose) [3] and a small blood sample was taken from the animals’ caudal vein through a small perforation, with a number 11 scalpel slide. The blood sample should be a compatible drop for reading the glucose-meter. The results presented are the average obtained in each group in the periods evaluated.

### 2.6. Sample Processing

Animals were euthanized at 3 (*n* = 4 by group), 7 (*n* = 4 by group), 14 (*n* = 6 by group) and 42 (*n* = 10 by group) days after right upper incisor extraction (Figure 1), with high dose of anesthetic solution infiltration. The jaws collected at 3, 7, 14 and 42 days were decalcified in EDTA (10%), for later inclusion in paraffin to obtain cuts 5 μm thick, mounted on slides. The histological slides were separated for the staining of picrosirius-red and immunohistochemical reactions. Part of the jaws collected at 42 days were stored in 70% alcohol to analysis of computerized digital microtomography system, and then were processed to obtain the sections for confocal microscopy. It is important to mention that the area of interest in all analyses was the alveolar middle third.

### 2.7. Microtomographic Analysis (Micro-CT)

The samples were scanned by the SkyScan microtomograph (SkyScan 1176 BrukerMicroCT, Aatselaar, Belgium) using cuts of 9 μm thickness (50 Kv and 500 μA), with a copper and aluminum filter and rotation step of 0.3 mm. The images obtained by X-ray projection in the samples were reconstituted by the software NRecon (SkyScan, 2011; Version 1.6.6.0, Edinburgh, UK), determining the area of interest. In the Data Viewer software (SkyScan, Version 1.4.4 64-bit, Edinburgh, UK), the images were reconstructed to fit the standard positioning for all samples, and can be observed in three planes: transverse, longitudinal and sagittal. Then, the software CTAnalyser—CTAn (2003-11SkyScan, 2012 BrukerMicroCT Version 1.12.4.0, Edinburgh, UK) defined the area of interest for three-dimensional evaluation (ROI). The CTAn software analyzes and measures images according to the gray scale (threshold). The threshold used in the analysis will be 25–90 shades of gray, which makes it possible to obtain the volume of bone formed in the tooth socket under repair [29].

The images obtained from the computerized microtomography were used to characterize the mineral bone density in each of the experimental groups and percentage of bone volume (BV/TV), trabecular thickness (Tb.Th), as well as the distance between the trabeculae (Tb.Sp) and trabecular number (Tb.N) [29]. Also was used the parameters of bone surface (BS) and bone porosity (Po.Tot) [26].

### 2.8. Immunolabeling Analysis

The immunohistochemical processing was performed in the Laboratory for the Study of Mineralized Tissues, Department of Basic Sciences, Araçatuba Dental School—São Paulo State University (São Paulo Research Foundation—FAPESP—2015/14688-0).

The primary antibodies (Santa Cruz Biotechnology, Inc., Dallas, TX, USA) were used against tumor necrosis factor alpha—TNFα (SC1070), Transforming Growth Factor Beta—TGFβ (SC1238) and interleukin 6—IL-6 (SC1265) to observe inflammatory responses at 3 and 7 days after dental extraction. With the slices obtained by 14 and 42 days after dental extraction were used antibodies against Wnt signaling pathways—WNT (SC69135), osteocalcin—OC (SC18319) and tartrate-resistant acid phosphatase—TRAP (SC30832) to analyze the process of bone formation and bone resorption. The analysis was performed with an optical microscope (LeicaR DMLB, Heerbrugg, Switzerland) by means of scores (ordinal qualitative analysis); when the scores were light labeling (++), moderate labeling (+++), and intense labeling (++++), it was considered positive for diaminobenzidine, taking care to hold negative controls to evaluate the specificity of the antibodies. These scores were established according to previous studies [26,30,31], where light labeling represented about 25% of immunolabeling area in the blades, moderate labeling represented about 50%, and intense labeling represented about 75%.

### 2.9. Collagen Fibers Maturation (Birefringence Analysis)

Histological slices of 14 and 42 postoperative days, were stained with Picrosirius red and analyzed under a polarized light microscope in order to evaluate the maturation, organization and number of collagen fibers, considering the birefringence intensity of the collagenous matrix [32]. Quantification of birefringence brightness intensity was performed using ImageJ software (Image Processing and Analysis Software, Toronto, ON, Canada). Initially, the green, yellow, orange and red color spectra were defined following RGB values (red, green, blue), standardized for all images. For quantification, the images were binarized for each color spectrum, as well as the amount in pixels of each color corresponding to each field. Subsequently, the averages of the values in pixels were calculated and subjected to statistical analysis in order to calculate the means and standard deviations of each group in each experimental period. Birefringent collagen fibers are showed in green and red shades, representing a less organized and immature collagen matrix, and a more organized and mature collagen matrix, respectively.

### 2.10. Confocal Microscopy Analysis

After the microtomography, the same samples were dehydrated in increasing concentrations of alcohols, then imbibed and infiltrated in a 1:1 solution of acetone and slow methyl methacrylate (PMMAL) (Classical, Classical Dental Articles, São Paulo, Brazil). Subsequently, they were given 3 PMMAL baths, and in the last bath, the 1% benzoyl peroxide catalyst (Riedel—De Haën AG, Seelze—Hannover, Germany) was added. The latter bath (PMMAL and catalyst) was performed with the pieces placed in capped glass vials, which were held at a temperature of 37 °C for 5 days until the resin was polymerized. After polymerization, the blocks containing the specimens were initially reduced with a “Maxcut” drill mounted on a Kota bench motor (Strong 210, São Paulo, Brazil), parallel to the long axis of the hemimaxilla (sagittal plane). Progressive manual wear on an automatic polisher (ECOMET 250PRO/AUTOMET 250, Buehler, Lake Bluff, IL, USA) was used. The sections were mounted on histological slides. After that, the sections were analyzed at Leica CTR 4000 CS SPE microscope (Leica Microsystems, Heidelberg, Germany) under a 10x objective (original magnification 100). The images were reconstructed, and the alveolar bone presented the overlap of the two fluorochromes [33]. These images were exported to ImageJ software (Image Processing and Analysis Software) to measure the bone dynamics [27] represented by the difference between old bone and new bone and active surface of mineralization [26] by the higher prevalence of red alizarin.

### 2.11. Statistical Analysis

For the data statistics, the area of bone tissue presents in the middle third of socket was evaluated. In order to compare the values obtained in the different groups and experimental periods, the normality test (Shapiro Wilk) was first performed; according to the distribution of quantitative results in the normality curve, a parametric test was chosen (unpaired T test), and the statistical program GraphPad Prism 7.03 (GraphPad Software, San Diego, CA, USA). A significance level of *p* < 0.05 was adopted.

## 3. Results

### 3.1. Glycose Level Is Increased after Cafeteria Diet and STZ Application in T2D

**Figure 2:**
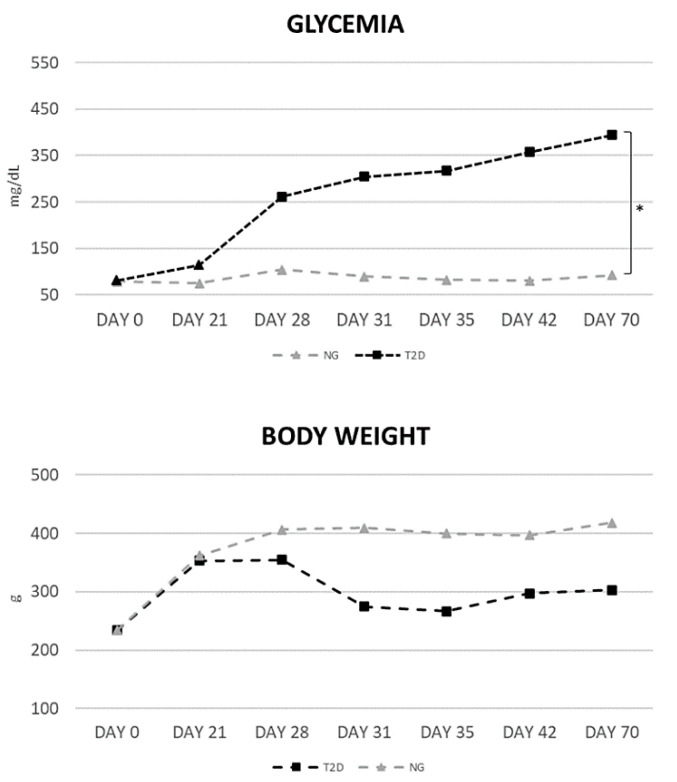
Graphs of Glycemia and Body Weight averages at beginning of diet, three weeks of diet, diabetes confirmation/dental extraction and euthanasia. Glycemia: Graph of the glycemic index monitoring over the days. Type 2 diabetic animals have a higher glycemic index, with statistically significant difference when compared to the normoglycemic group. Body Weight: Graph of the body weight monitoring over the days. Type 2 diabetic animals have a weight decrease, with no statistically significant difference when compared to the normoglycemic group. Symbols: * *p* < 0.05.

At day 0, the animals present a normal average glycose level, similar between the groups. Three weeks after the beginning of the cafeteria diet, the T2D glycose level increased with a statistically significant difference, remaining until the end of the experiment. In contrast, the blood glucose levels average in the control group remained constant until the end of the experiment, with values close to 80 mg/dL. There was a statistically significant difference between the groups and the periods analyzed, where T2D animals presented higher average of blood glucose levels (Figure 2 and Table 2). Both groups showed a statistically significant difference in the body weight average at the end of the experiment when compared to the initial period. CO group has a higher body weight average, but no statistically significant difference between the groups (Figure 2 and Table 3).

### 3.2. T2D Impairs the Alveolar Bone Quality as a Result of the Decrease in Bone Volume and Increase in Porosity

**Figure 3:**
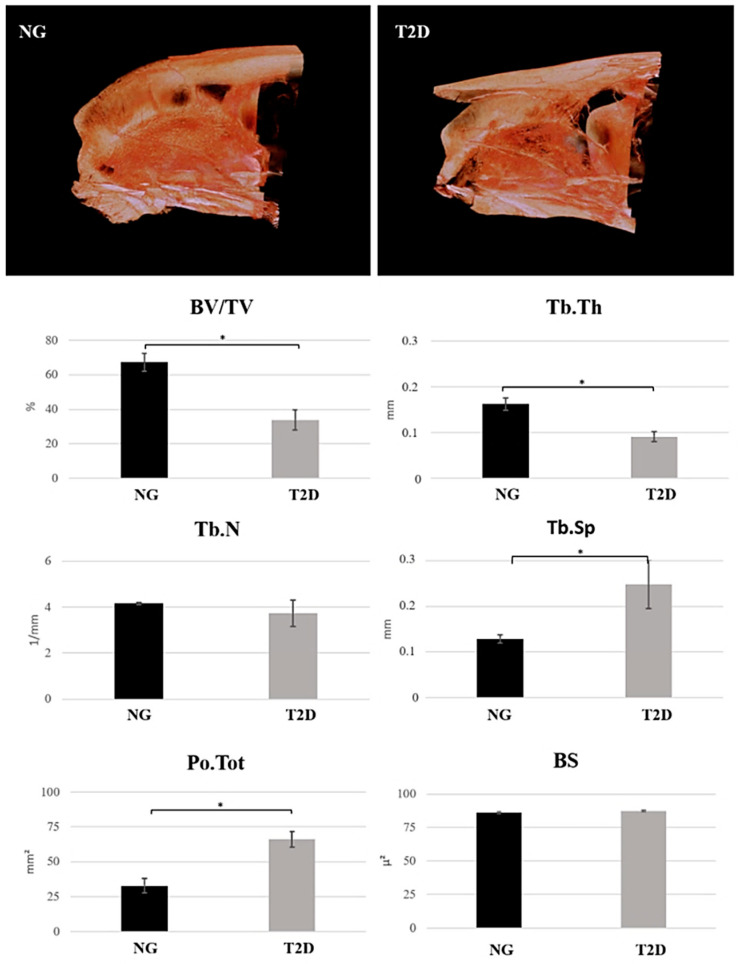
Scan and graphs showing Micro-CT results. Representative image of NG and T2D maxilla. BV/TV: T2D group have a lower numerical value of bone volume percentage when compared to the NG group, with statistically significant difference between the groups. Tb.Th: T2D group have a lower numerical value of trabecular thickness when compared to the T2D group with statistically significant difference between the groups. Tb.N: There is no statistically significant difference in the number of trabeculae between groups. Tb.Sp: T2D have a higher trabecular separation when compared to NG group, with statistically significant difference between the groups. Po.Tot: 2D have a total porosity when compared to NG group, with statistically significant difference between the groups. BS: There is no statistically significant difference in the bone surface area between groups. Symbols: Error bar; * *p* < 0.05.

BV/TV: For the parameter of bone volume percentage, there was a statistically significant difference between the groups (*p* = 0.0001). The NG group had a higher numerical value of bone volume percentage (BV/TV = 67.25%) when compared to the T2D group (BV/TV = 33.84%) (Figure 3).

Tb.Th: Thickness of trabecular bone in the dental socket of the NG group was higher (Tb.Th = 0.1618 mm) when compared to the T2D group (Tb.Th = 0.0909 mm), presenting a statistically significant difference (*p* = 0.0002) (Figure 3).

Tb.N: There was no statistically significant difference for this parameter (*p* = 0.1771). The numerical values obtained for the groups were similar (Figure 3).

Tb.Sp: Regarding the space presented between the trabeculae, the T2D group had a higher value (Tb.Sp = 0.2485 mm) when compared to the NG group (Tb.Sp = 0.1288 mm), presenting a statistically significant difference between the values (*p* = 0.045) (Figure 3).

Po.Tot: T2D group presented bone with a higher porosity (Po.Tot = 66.16%) when compared to the NG group (Po.Tot = 32.75%). There was a statistically significant difference between the groups (*p* = 0.0001) (Figure 3).

BS: There was no statistically significant difference between the groups (*p* > 0.05). The groups show similarity for the BS values (*p* = 0.4685) (Figure 3).

### 3.3. The Increasing Expression of Inflammatory Proteins Results in the Reduction of Proteins Responsible for Bone Formation and an Increase the Osteoclastic Activity in the Alveolar Bone

**Figure 4:**
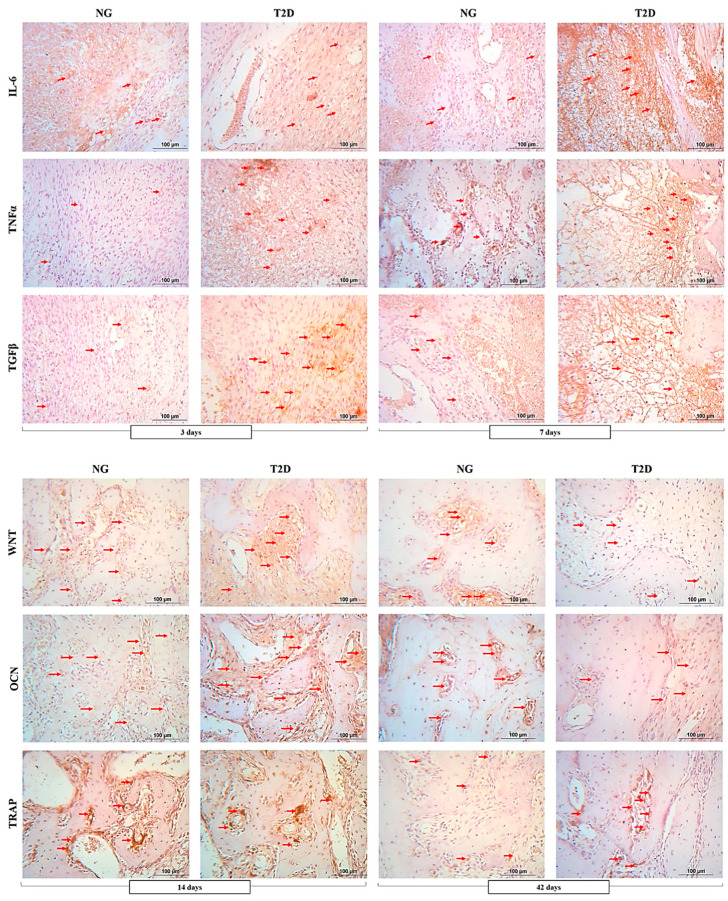
NG and T2D photomicrographs of IL-6, TNFα, TGFβ, OC, Wnt and TRAP immunolabeling. To observe inflammatory responses at 3 and 7 days after dental extraction, were evaluated the immunolabeling of IL-6, TNFα and TGFβ. To analyze the process of bone formation and bone resorption at 14 and 42 days after dental extraction was evaluated the immunolabeling of Wnt, OC and TRAP. The scores are shown in Table 3. Symbols: red arrows—Immunolabeled cells. Counterstaining: Harris hematoxylin. 25× magnification; 100 μm scale bar.

IL-6: At 3 days, the dental socket under repair in NG was observed with remnants of the blood clot and incipient granulation tissue, characteristic of this stage. Positive labeling for IL-6 was observed in inflammatory cells, covering a small area of the socket under repair (++). At 7 days, the NG connective tissue was organized, with few blood clot remnants, and it was possible to visualize bone trabeculae in the initial stage of formation. The labeling for IL-6 is thus shown to be light (++). The T2D group, has a tissue with a predominance of inflammatory cells—A marked characterization of this cytokine—Was observed in the socket under repair (++++) (Figure 4, Table 4).

TNFα: At 3 days, TNFα immunostaining, light positive labeling (++) of the inflammatory cells was observed in the socket under repair for the NG group. However, in the T2D group, moderate labeling (+++) was observed, extending throughout dental socket. At 7 days, observing the incipient organization of newformed trabecular bone in NG group, it was possible to observe light to moderate (++/+++) labeling in the inflammatory cells, present in a small quantity. T2D group, again highlighted the delay in the reparative events in relation to the NG group, noting the presence of granulation tissue with inflammatory infiltrate, moderately labeled for TNFα (+++) (Figure 4; Table 4).

TGFβ: At 3 days, light labeling (++) of TGFβ was observed for inflammatory cells in the dental socket of the NG group. In this same period, T2D group has moderate labeling (+++) of this same factor observed in the inflammatory cells of the socket under repair. At 7 days, still in relation to TGFβ, moderate labeling (+++) was observed in the inflammatory cells in the socket of NG group, and intense labeling (++++) in the inflammatory cells in the alveolar bone of T2D group (Figure 4; Table 4).

OC: At 14 days, was observed an organized trabecular bone in NG group, with osteoblasts presence in the activity of organic matrix synthesis next to the bone tissue. In this group, OC was moderately stained (+++) in both the form of precipitate on the newformed trabecular bone and on the osteoblasts in organic matrix synthesis activity. In the T2D group, a pattern of incipient bone trabeculation was observed, with thinner trabeculae and a large amount of connective tissue, moderately labeled (+++) for OC. Osteoblasts were also positively stained for this protein. At 42 days, the bone tissue in the NG group was already observed filling a large part of the alveolar bone under repair, with small marrow spaces, OC was moderate labeled (+++), showing mature bone tissue. T2D group showed a delay in these reparative responses, with light labeling (++) for OC in the same period, especially in connective tissue that fills an important portion of the socket under repair (Figure 4; Table 4).

Wnt: At 14 days, the Wnt protein in the NG group was moderately labeled (+++), especially in the osteoblasts next to the newly formed bone tissue. It was also shown to be moderate (+++) in the T2D group; however, it is worth noting that in this group, there was an important labeling in the connective tissue around the neoformed trabecular bone. At 42 days, Wnt protein was observed in NG ground next to the osteoblasts present in the bone marrow spaces of the neoformed trabecular bone, characterized by moderate labeling (+++). In T2D group, light labeling (++) was observed in osteoblasts and extracellular matrix of the bone tissue present in the socket under repair (Figure 4; Table 4).

TRAP: The osteoclastic activity in the dental socket under repair was moderately labeled (+++) for the NG group and T2D group at 14 days. This labeling shows metabolic activity of the alveolar bone in this stage of the repair process, characterizing activation of basic multicellular units in this period. At 42 days, it was already possible to observe light labeling (++) for TRAP in the NG alveolar bone, and moderate labeling (+++) for the same protein in the T2D group, which shows the activity of bone resorption maintained until the final stage of alveolar repair (Figure 4; Table 4).

### 3.4. T2D Decreases the Maturation of Collagen Fibers during the Alveolar Bone Repair Process

**Figure 5:**
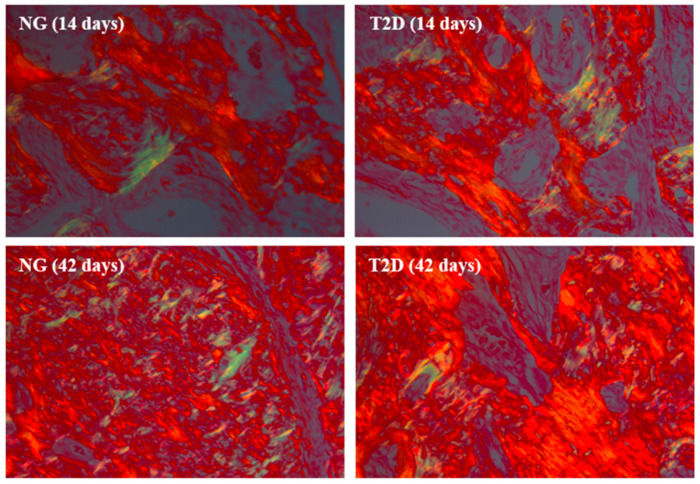
Collagen maturation photomicrographs. Photomicrographs at 14 and 42 days after dental extraction obtained by a polarized microscopy, showing birefringent collagen fibers in the green and red shades, representing a less organized and immature collagen matrix and a more organized mature collagen matrix, respectively. Counterstaining: Picrosirius red. Original 25× magnification.

**Figure 6:**
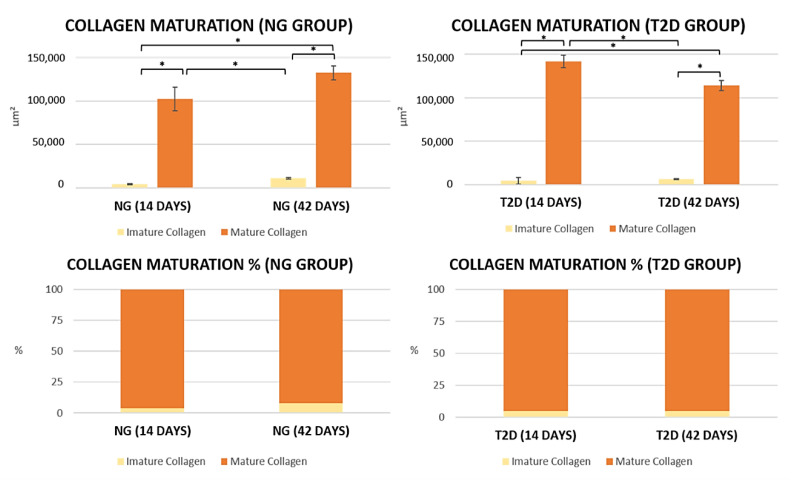
NG and T2D collagen fibers maturation. Graphs showing collagen maturation between the groups. It is possible to observe that the T2D group has a decrease in mature collagen fibers over the days, while the NG group has an increase in mature collagen fibers. Symbols: Error bar; * *p* < 0.05.

The results of collagen fiber maturation showed that at 14 days, the NG group presents a lower total amount of collagen fibers when compared to the T2D group. At 42 days after tooth extraction, the T2D group showed a decrease in the amount of collagen fibers in the dental socket, while the T2D group showed a decrease in the total amount of fibers (Figure 5 and Figure 6).

In the percentage graph of collagen fibers, it is possible to observe that both groups present similarity for the percentages of mature and immature collagen, however, it is important to stress again that the total amount of collagen fibers on T2D is decreased with the passing of the days of alveolar repair. Numerical values for the quantification of birefringent fibers are shown in the graphs (Figure 6), showing the comparison between groups NG and T2D at 14 and 42 days.

### 3.5. T2D Compromises Bone Turnover after Tooth Extraction

**Figure 7:**
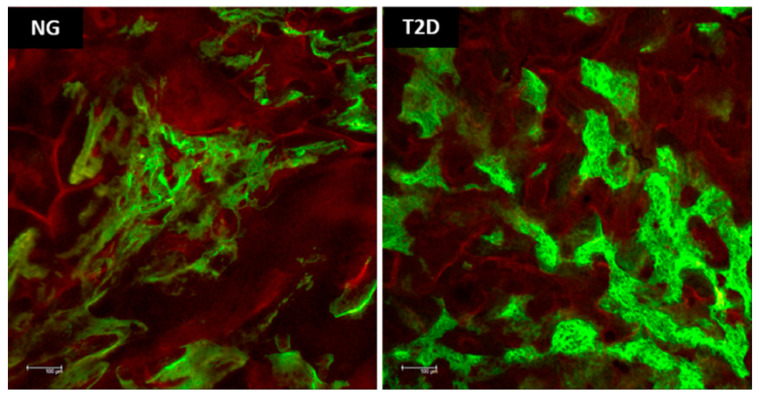
Confocal microscopy photomigrographs. The photomicrographs obtained by a confocal microscopy, show the fluorochromes precipitation. In green: Calcein precipitation in the bone matrix; In red: Alizarin red precipitation in the bone matrix. Original 10× magnification.

Qualitative analysis of fluorochromes [34]: As mentioned, two intramuscular injections of fluorochromes were applied at different times, making it possible to analyze the dynamics of mineral matrix deposition in a period closer to dental extraction and in a later period. The T2D group presents a larger area of precipitation of calcein, indicating a greater amount of old bone when compared to the NG group. The area of alizarin precipitation in T2D group is smaller, indicating a lower deposition of new mineral matrix in this group when compared to NG (Figure 7).

**Figure 8:**
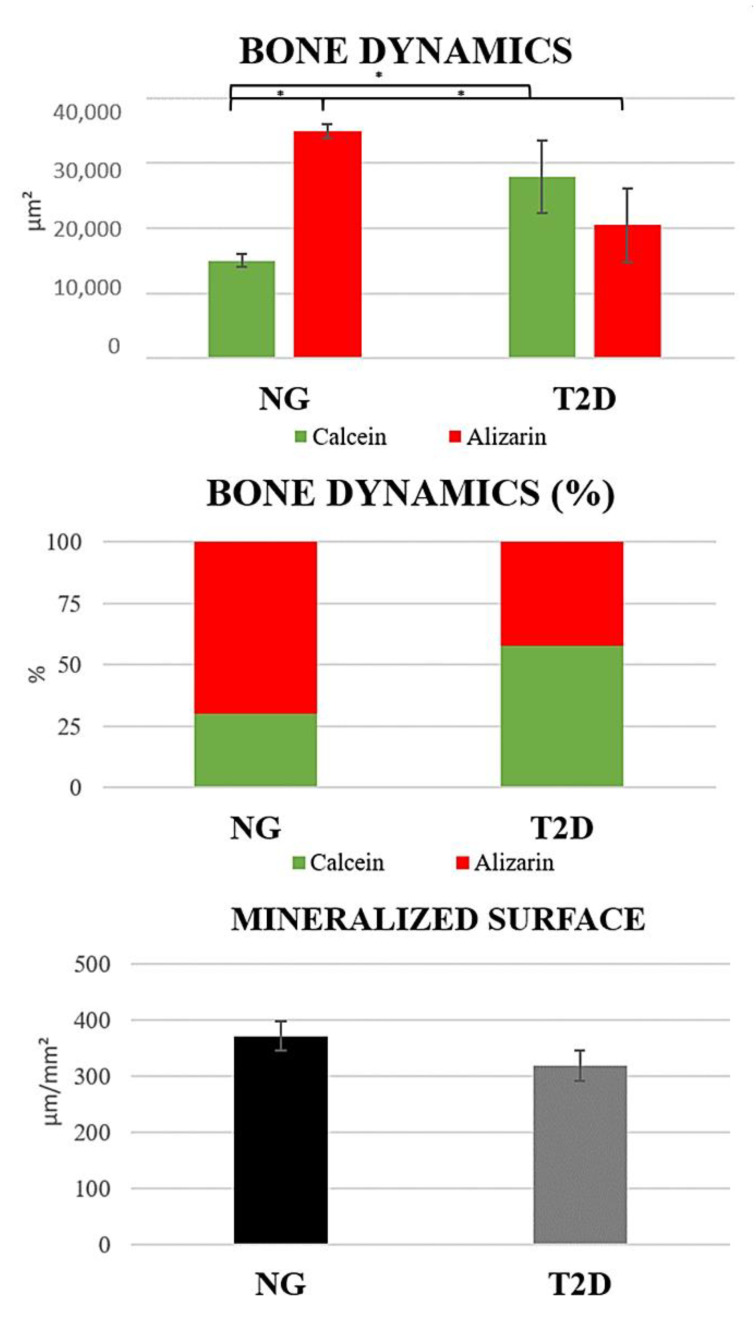
Confocal microscopy results. Bone Dynamics: The graphs show a statistically significant difference between the fluorochrome precipitations, where T2D presents a lower percentage of new bone and a higher percentage of old bone. Mineralized surface: There is no statistically significant difference between the mineralized surface area. Symbols: Error bar; * *p* < 0.05.

Bone dynamics: Showed a statistically significant difference between all fluorochrome precipitations (*p* < 0.05), in the percentage area of fluorochromes graph it is possible to observe the inversion of the values between the groups, where T2D presents a lower percentage of new bone and a higher percentage of old bone (Figure 8).

Mineralized Surface: There was no statistically significant difference between the groups (*p* = 0.5849), where T2D shows smaller mineralized area (MS = 318.4 µ^2^) when compared to NG (MS = 371.4 µ^2^) (Figure 8).

## 4. Discussion

Studies about bone repair and type 2 diabetes show that long bones suffer with the impact of diabetes [20,35,36], especially type 1 where there is a lack of insulin which has a role as an anabolic substance affecting bone [8]. The purpose of this paper was to study a more specific bone tissue, considering that its alveolar repair process happens differently from long bones [25].

Correlating the development of that systemic condition in humans with the animal model, this study showed that the cafeteria diet increases the glycemic patterns of the animals at the end of the initial three weeks, similar to what happens in the population using poor nutrition diet. Streptozotocin assumes a role in advancing the process of pancreatic beta cell degradation [3,12,13,14] in this model in a partial way, thus decreasing the time for a diagnosis of type 2 diabetes. It is important to emphasize that hyperglycemia, weight loss, polydipsia, polyphagia, polyurea and blurred vision are factors that together bring severe complications to untreated diabetic patients [1,2]. The blood glucose results show that this model becomes viable, and the animals present an altered glycemic pattern when compared to the normoglycemic group, extending until the moment of euthanasia. Even without statistical difference, there was a weight decrease of T2D animals after streptozotocin injection, which would be directly related to drug application, as well as acute hyperglycemia [3]. Besides hyperglycemia, undiagnosed and untreated diabetic patients present constant polyuria and weight loss [5,10,11,12,13], characteristics also observed in the animals of this study. These effects are presented after the application of STZ, where degradation of beta cells occurs, showing once again the effectiveness of the drug in type 2 diabetes animal models.

The impact of diabetes is observed in several body tissues [6] and bone is also affected [35,36,37], decreasing quality and increasing the risk of fracture [38]. This aspect can be confirmed on 3D microtomography results, performed in the present study, that shows an impairment of bone repair quality of type 2 diabetic animals in comparison with normoglycemic. The evaluation of parameters related to trabeculae characteristics confirm this finding and show that the bone formed during the repair process after upper incisor extraction suffer an impairment of bone formation activity. Therefore, after the dental extraction, type 2 diabetes animals have a bone volume at half the value presented in normoglycemic animals at 42 days, as well as a higher total porosity. These data show that even with a similar alveolar bone surface, type 2 diabetes shows a loss in alveolar bone quality and quantity when compared to normoglycemic animals.

Studies comparing the bone microarchitecture from diabetic rats and normoglycemic rats showed that long bones and mandibular bone respond negatively in the presence of diabetes mellitus [39]. However, more specialized tissues such as the alveolar bone were not widely studied in type 2 diabetes. Micro-CT results show that bone microarchitecture in the final step of alveolar repair process and the other analysis performed in this study reveal the influence of the type 2 diabetes condition in the mineralization process observed in the alveolar repair.

Inflammatory process is important in the initial period of the alveolar repair, as long as it occurs in a mild manner, decreasing with the passing of days [25,40]. IL-6, TNFα and TGFβ command tissue inflammatory responses, playing an important role in the start of the repair process [23,41]. IL-6 have been associated with regulatory roles in bone remodeling, TNF-α has a synergistic effect on RANKL-induced osteoclastogenesis and TGFβ is involved in the degradation of the extracellular matrix [42]. Immunohistochemical analysis showed that at 3 days after dental extraction, both groups presented inflammatory protein expression in a similar mode, but type 2 diabetes stimulated the inflammatory process in the alveolar bone at 7 days after dental extraction when compared to the normoglycemic group. Inflammatory environments and chronic hyperglycemia in T2D affect factors that regulate bone remodeling, such as increasing the levels of advanced glycation end products (EGAs), which directly affect the bone matrix and influence bone cells through the formation of reactive oxygen species [42,43].

After the inflammatory process, the maturation of the collagen fibers in the bone tissue is initiated [25,40]. This stage of alveolar repair assumes great importance at the beginning of the bone mineralization process [25,40], due to the collagen providing a scaffold on which hydroxyapatite crystals can mineralize, thus contributing to bone strength, tensility and structural integrity [42]. The results that show the maturation and organization of collagen fibers show that the type 2 diabetes group has a decrease in the total amount of collagen fibers along the alveolar repair, with deposition of these fibers in a disorganized manner, when compared to normoglycemic animals. When comparing the percentage of mature and immature collagen it is possible to note that there is no difference between the groups.

In conditions of hyperglycemia, collagen glycosylation and the accumulation of AGEs occur, as a result, the collagen is more rigid and of worse quality, reducing the flexibility of T2D bones [42]. The decrease of collagen fibers in the post-exodontic socket has an impact on the mineralization process, so the immunohistochemical analysis performed in the periods of 14 and 42 days after dental extraction shows the process of mineralization and bone resorption. Wnt is responsible for stimulating calcium release in bone tissue, while osteocalcin is expressed at the end of the mineralization process, and TRAP shows osteoclasts in resorption activity [30,31]. The NG group presents moderate immunolabeling for Wnt and osteocalcin proteins at both 14 and 42 days, and presents a decrease in the bone resorption process at 42 post-operative days. In contrast, the type 2 diabetes group shows a decrease in the bone formation process and an increase in the resorption process at 42 days, showing an imbalance and delay in the alveolar repair process. Thus, when organism suffer a trauma, for example, tooth extraction surgery, there is a condition of hypermetabolism that is developed in order to compensate the tissue repair responses [40].

Another important aspect that should be taken into consideration in the alveolar bone repair is the dynamics of bone tissue. The fluorochromes precipitation (calcein × alizarin) in the bone matrix facilitates the analysis at two different times of the repair process [26,27,28]. The results of this analysis showed that the type 2 diabetes has a loss in bone remodeling process because it presents a larger area of bone precipitated by calcein (old bone), while the normoglycemic group presents a larger area precipitated by alizarin (new bone). When analyzing the area percentages of each fluorochrome there is an inversion of the proportion of these values, indicating an impact on bone turnover of type 2 diabetes. This result corroborates the findings of immunohistochemical analysis and collagen fiber maturation that were shown to be impaired in the type 2 diabetes. The active mineralization surface did not present statistical difference between the groups, but it is important to note that the precipitation of alizarin is lower in the type 2 diabetes and both groups presented a similar value of bone surface area, which would explain this result.

The scientific evidence presented in this study shows that type 2 diabetes prolongs the local inflammatory process, which impairs the organization and maturation of collagen fibers, delaying bone formation that generates impact on mineralization and bone turnover. This fact implies in a series of disorders in dental practice, such as the need for dental extractions and rehabilitation with dental implants that would need to compensate in other ways, either with systemic medications or local therapies in the alveolar bone under repair. As this is the first study of our group where the alveolar bone repair is evaluated in type 2 condition, the idea is to characterize the steps of alveolar repair process in this condition and in a second moment, evaluate the therapeutical strategies that can improve the characteristics of the repair bone formed into the extraction socket. More studies will be developed in this direction, considering the alveolar repair as well as implant’s installation models.

## 5. Conclusions

It is possible to conclude that type 2 diabetes triggers a sequence of events that are responsible for the delay of the alveolar repair process in comparison to normoglycemic conditions. In addition, the inflammatory response in the initial stage of alveolar repair process is responsible for the impairment of collagen fiber organization as well as the normal mineralization process, resulting in poor-quality bone filling at dental sockets. Finally, the reduced participation of the Wnt pathway as well as the role of bone matrix proteins like osteocalcin contribute to the impairment of bone repair quality.

## Figures and Tables

**Table 1:** Cafeteria Diet.

Foods	Amount (per Rat)
Stuffed Crackers	30 g daily
Wafer Crackers	30 g daily
Corn Chips	30 g daily
Water with Sugar (12%)	50 mL daily

Foods that make up the cafeteria diet (T2D): Stuffed crackers, wafer crackers, corn chips and also a bottle of water with a concentration of 12% of sucrose.

**Table 2:** NG and T2D glycemia average at evaluated periods.

Group	Day 0	Day 21	Day 28 *	Day 31 *	Day 35 *	Day 42 *	Day 70 *
NG	79 mg/dL	75 mg/dL	104 mg/dL	90 mg/dL	82 mg/dL	80 mg/dL	92 mg/dL
T2D	80 mg/dL	114 mg/dL	262 mg/dL	305 mg/dL	317 mg/dL	358 mg/dL	394 mg/dL

Beginning of the diet = day 0; STZ application = day 21; Diabetes confirmation/dental extraction = day 28; and euthanasia = at days 31, 35, 42 and 70. * There was statistical difference between the groups.

**Table 3:** NG and T2D body weight average at evaluated periods.

Group	Day 0	Day 21	Day 28	Day 31	Day 35	Day 42	Day 70
NG	234 g	362 g	406 g	408 g	400 g	397 g	418 g
T2D	234 g	353 g	355 g	275 g	267 g	297 g	303 g

Beginning of the diet = day 0; STZ application = day 21; Diabetes confirmation/dental extraction = day 28; and euthanasia = at days 31, 35, 42 and 70. There was no statistical difference between the groups.

**Table 4:** Scores of NG and T2D immunolabeling analysis. Antibodies against IL-6, TNFα, TGFβ, OC, Wnt and TRAP.

Group	Protein	Score	Days
NG	IL-6	++	3 Days
		++	7 Days
	TNFα	++	3 Days
		++	7 Days
	TGFβ	++	3 Days
		+++	7 Days
	OC	+++	14 Days
		+++	42 Days
	Wnt	+++	14 Days
		+++	42 Days
	TRAP	+++	14 Days
		++	42 Days
T2D	IL-6	+++	3 Days
		++++	7 Days
	TNFα	+++	3 Days
		+++	7 Days
	TGFβ	+++	3 Days
		++++	7 Days
	OC	+++	14 Days
		++	42 Days
	Wnt	+++	14 Days
		++	42 Days
	TRAP	+++	14 Days
		+++	42 Days

Light labeling (++), moderate labeling (+++), and intense labeling (++++).
